# Direct costs of dementia in nursing homes

**DOI:** 10.3389/fnagi.2015.00146

**Published:** 2015-07-28

**Authors:** Hilma Caravau, Ignacio Martín

**Affiliations:** ^1^Institute of Electronics and Telematics Engineering of Aveiro, University of AveiroAveiro, Portugal; ^2^Health Sciences Department, CINTESIS (UNIFAI. UA), University of AveiroAveiro, Portugal

**Keywords:** dementia, direct costs, nursing homes, economics, cost of illness, Portugal

## Abstract

Dementia represents an economical burden to societies nowadays. Total dementia expenses are calculated by the sum of direct and indirect costs. Through the stages of the diseases, as the patients may require institutionalization or a formal caregiver, the direct costs tend to increase. This study aims to analyze the direct costs of dementia in Portuguese nursing homes in 2012, compare the spending between seniors with and without dementia, and propose a predictive costs model. The expenses analysis was based on (1) the use of emergency rooms and doctor's appointments, either in public or private institutions; (2) days of hospitalization; (3) medication; (4) social services use; (5) the need for technical support; and (6) the utilization of rehabilitation services. The sample was composed of 72 people, half with dementia and half without. The average annual expense of a patient with dementia was €15,287 thousand, while the cost of a patient without dementia was about €12,289 thousand. The variables “ability to make yourself understood,” “self-performance: getting dressed” and “thyroid disorders” were found to be statistically significant in predicting the expenses' increase. In nursing homes, in 2012, the costs per patient with dementia were 1, 2 times higher than per patient without dementia.

## Introduction

Dementia encompasses a number of diseases and conditions with specific characteristics, such as cognitive impairment, functional, and behavioral (Reitz et al., 2011), and is a major cause of disability in old age (World Health Organization and Alzheimer's Disease International, 2012).

In 2010, the number of people with dementia in the world was estimated at 35.6 million. It is expected that the number will reach 65.7 million in 2030 and 115.4 million in 2050 (Ferri et al., 2005; World Health Organization and Alzheimer's Disease International, 2012).

The economic impact of dementia in society is connected with the resources used to prevent, diagnose, treat, rehabilitate, and cope with the illness (Huang et al., 1988; O'Shea and O'Reilly, 2000).

The total cost of dementia is produced by finding the sum of two types of costs, direct and indirect (Jönsson, 2004; Castro et al., 2010).

Indirect costs represent lost resources. These relate to the loss or reduction of earnings by the patient and/or family members arising from the lost or reduced productivity associated with premature death or absenteeism from work due to dementia (Jönsson, 2004; Castro et al., 2010).

The direct costs represent the resources used (Wimo et al., 2007), which tend to increase with the progression of the disease, with institutionalization, or when there is a need for a caregiver. The costs of these resources are evaluated directly from the market value of goods and services (Huang et al., 1988). Direct costs include all of the costs directly accountable, from the medical and non-medical sector, which include hospital stays, medical services, medical drugs, social services, rehabilitation services, and formal payments to family caregivers (Jönsson, 2004; Castro et al., 2010). Institutionalization converts indirect costs into direct ones (Castro et al., 2010).

In 2008, the EuroCoDe project, considering 7.23 million people with dementia, estimated that the total cost of dementia in the European Union 27 (EU27) was around €160 billion (€22,000 per patient per year). Out of this amount, 56% of the cases were attributed to informal care costs and the remaining 44% to direct costs (Wimo et al., 2011).

The direct costs of dementia care were estimated to be US$279 billion when looking at 34.4 million cases of dementia in 2009 (Wimo et al., 2010).

In Portugal, in 2005, there were estimated to be approximately 113,039 people with dementia representing a direct cost of US$952.9 million (Wimo et al., 2007). In 2009, out of 142,790 diagnosed cases of dementia, the direct costs increased to US$1296.5 million (Wimo et al., 2010).

This study aims to analyze the direct costs of dementia in Portuguese nursing homes in 2012, compare the spending between seniors with and without dementia, and propose a predictive costs model using the selected variables of the Resident Assessment Instrument-Resource Utilization Group (RAI-RUG) (Gray et al., 2008). The RAI-RUG is one of the Quality Control System tools of gerontology, and it is considered the most complex one of them all. This protocol is a document of the Unit for Research and Education on Adults and Elders (UNIFAI).

## Methods

### Subjects

Firstly, by convenience and through the technique of non-probability sample (Ritchie and Lewis, 2003), four non-profit organizations that fell within the requirements of the RAI-RUG were included. In these four institutions, all the subjects selected were in nursing homes with a clinical diagnosis and signs of dementia.

The selection of subjects with signs of dementia was made by the nursing staff and the technical director of the institutions.

The diagnosis of dementia is a complicated process, takes a long time and carries a high economic cost (Zhu and Sano, 2006). For this reason, a several number of subjects had several signs of dementia (memory loss, disorientation, confusion, personality changes, among others World Health Organization and Alzheimer's Disease International, 2012), understood by health workers (nurses and doctors) but did not have a diagnosis definitive. Whereas a number of subjects would be left out of the study for not having a clinical diagnosis written, and in the presence of consensus between the team of PISS, these users were incorporated into the study. These signs have been checked by the team over the daily routines of the subject and in conversation with the elements of direct relationship of the person, such as family.

An equal number of subjects without dementia were included through the random paired sampling technique (Datallo, 2010). The sample was composed of 72 people, half with dementia and half without. Socio-demographic information of the sample can be seen in Table 1. Clients who were admitted after the evaluation of RAI-RUG were not included.

**Table 1:** **Socio-demographic information of the sample (***n*** = 72) divided into two groups: with dementia (***n*** = 36) and without dementia (***n*** = 36)**.

**Variable**	**With dementia (*n* = 36)**	**Without dementia (*n* = 36)**	**Chi-square**	***p*-value**
	**n (%)**	**n (%)**		
**GENDER**				
Male	6 (16.7)	19 (52.8)	10.356	0.001
Female	30 (83.3)	17 (47.2)		
**AGE**				
65–74	1 (2.8)	7 (19.4)	5.063	0.080
75–84	18 (50)	15 (41.7)		
> 84	17 (47.2)	14 (38.9)		
**SCHOOLING**				
< primary	24 (66.7)	23 (63.9)	0.061	0.804
≥primary	12 (33.3)	13 (36.1)		
**MARITAL STATUS**				
Single	5 (13.9)	8 (22.2)	1.969	0.579
Married	7 (19.4)	6 (16.7)		
Widowed	24 (66.7)	21 (58.3)		
Divorced	0 (0)	1 (2.8)		
**HOSPITALIZATION TIME**				
< 1 year	20 (55.6)	21 (58.3)	0.072	0.965
1–5 years	11 (30.6)	10 (27.8)		
>5 years	5 (13.9)	5 (13.9)		
**AMOUNT RECEIVED BY THE OWNER OF SOCIAL SECURITY**				
<€445	20 (55.6)	28 (77.8)	4.000	0.046
≥€445	16 (44.4)	8 (22.2)		
**STATE SEVERITY BIMS**				
Intact cognition	1 (2.8)	18 (50)	27.300	0.000
Moderate	4 (11.1)	8 (22.2)		
Severe	31 (86.1)	10 (27.8)		

An informed consent was not signed because we did not have access to personal information or identification. Authorization was requested to charge each institution for accessing the documents required for data collection.

Analyzing the results it turns out that people included in the group without dementia, at Brief Interview of Mental State (BIMS) show changes that put them in the level of moderate or severe impairment.

Cognitive changes may be associated with dementia or normal aging process. It was found that two-thirds of his sample had clinically definable cognitive impairment but they had no dementia (Graham et al., 1997).

This is a fact that happens repeatedly in nursing homes, and although in the study about 41.8% of the sample long-term care institutions showed cognitive impairment but not dementia. In their community sample, 46.3% of the subjects showed positive results for cognitive impairment (score < 78 in Modified Mini-Mental State Examination) but they have the diagnostic category on clinical assessment of “cognitive impairment, no dementia” (CIDN).

### Instruments

To assess the costs associated with dementia there are internationally defined protocols that include multiple dimensions.

These protocols are standardized into six dimensions, namely, (1) the use of emergency rooms and doctor's appointments, either in public or private institutions; (2) days of hospitalization; (3) medication; (4) social services use; (5) the need for technical support; and (6) the utilization of rehabilitation services (Ostbye and Crosse, 1994; Jönsson, 2004; Wimo et al., 2010). These were the six dimensions analyzed in this study. The RAI-RUG, indirectly, is an instrument used in this study.

### Procedures

Data collection took place between February and May 2013. Data was collected retrospectively in a period of 12 months (January 2012 to January 2013), through access to institutional documents, including daily occurrences to account for the number of trips to the emergency room, appointments in public or private institutions, hospital stay days, and consultations in rehabilitation services. The cost of six dimensions were analyzed.

#### Emergency rooms or appointments, in public or private institutions

In this dimension, there was found the number of times each user attended emergency department, hospital visits and consultations at the health center. Consultations in private hospitals were also considered. The calculation of each episode was done by multiplying the number of emergency episodes/consultations with the actual price of each episode. Financial, values for each episode were obtained considering the actual prices of health care episodes provided under the National Health Service^1^. Each basic emergency episode was considered to have the value of €51. Medical consultations in hospitals or health centers have a cost of €31. Private consultations represent a real cost of €80.

#### Days of hospitalization

To analyze the hospitalization costs, the number of days that each user was hospitalized in 2012 was recorded. These data were multiplied by the daily price of admission, a value of €85^1^.

#### Medication

The value that the medication represents for the patient was analyzed through access to monthly medication receipts. It was considered the charged value. These receipts are issued independently of costs to the inpatient service in nursing home.

#### Social services used

All subjects included in the study were part of the social response nursing home. For the assessment of this dimension, the reference value with families in nursing home for the year 2012 was considered. This amount is €930.06/user/month (Ministry of Solidarity Employment and Social Security and Union of Portuguese Mutual Societies, 2011).

#### Technical aids

The technical aids that each person used were checked with the staff and their price was assessed on commercial sites. The price of all technical aids used were considered. For technical aids with a long duration (such as beds and mattresses), however, we considered an amortization period of 8 years. To account for the cost of medical drugs and diapers (included under technical aids), the monthly bills of the subjects were acceded.

#### Rehabilitation services

The number of sessions of physiotherapy, occupational therapy, podiatry or chiropractic treatments and the assistive devices used by the patients were checked in the records or with the institution's staff. Differentiation was made between the prices of physical therapy sessions in relation to the severity of dementia according to the stages defined by the BIMS in RAI-RUG. In this test, higher scores indicate cognitively intact and lower scores show severe impairment (Department of Health and Mental Hygiene, 2011).

With the results of this test, it was possible to classify subjects with three scores: (1) intact cognition, (2) moderate, or (3) severe impairment (Department of Health and Mental Hygiene, 2011).

It is considered that a physiotherapy session consists of three phases, (1) heating step, (2) training phase, and (3) relaxation phase. Using the assumptions presented above, a set of treatments that should be included in a physiotherapy session at each stage of dementia and for subjects without dementia were defined. The prices for each technique are the prices set by the National Health Service^1^. The value of a physical therapy session for a user without dementia was estimated at €26.40, €49.6 for the slight stage and €54.4 for the severe stage.

Because this is a relatively cheap service when compared with a doctor's appointment and its price is not well established, by generalizing the values of occupational therapy, it is considered that a physiotherapy consultation costs €22. For occupational therapy sessions a value of €22 per session was considered, regardless of the level of severity^1^. For sessions of podiatry and chiropractic the values that the user disbursed in private services were counted, as these treatments are not included in the National Health Service price list.

### Statistical analysis

Statistical analyses were performed using the program IBM SPSS version 19.0. The significance level α = 0.05 was considered. Frequencies (absolute and relative) were used to provide descriptive statistics of the socio-demographic variables. Means and Standard Deviation (SD) were used to analyze the data dimension of economic costs. Univariable and multivariable general linear models were performed in order to assess associations between dementia costs and the characteristics of the subjects (RAI-RUG variables).

Were initially considered 400 variables of RAI-RUG in the model construction. Of all the variables included in the analysis, the total score was considered. Were included questions of these groups: (A) Identification Information (i.e., age, gender); (B) Hearing, Speech, and Vision (i.e., speech clarity); (C) Cognitive Patterns (i.e., temporal orientation); (D) Mood (i.e., Resident Mood Interview); (E) Behavior (i.e., psychosis); (G) Functional Status (i.e., bed mobility); (H) Bladder and Bowel (i.e., bowel continence); (I) Active Disease Diagnosis (i.e., heart/circulation, gastrointestinal, infections); (J) Health Conditions (i.e., pain management); (K) Swallowing/Nutritional Status (i.e., swallowing disorder); (L) Oral/Dental Status (i.e., broken or loosely fitting full or partial denture); (M) Skin Conditions (i.e., determination of pressure ulcer risk); (N) Medications (i.e., injections, insulin); (O) Special Treatments and Procedures (i.e., chemotherapy, dialysis); (P) Restrains (i.e., bed rall, trunk restraint) (Centers for Medicare and Medicaid Services, 2013). Pearson correlation was calculated with the objective of selecting the variables of RAI-RUG with the highest correlation with regard to the dependent variable (costs), being considered all variables with *p* < 0.05. Univariable logistic models were performed considering costs as dependent variables and each of the variables was significantly associated with the costs (26) found in the previous step. As a candidate for the multivariable model, we considered all variables significant at the 0.10 significance level in the univariable models. The final model includes all variables with *p* < 0.05.

## Results

### Costs dimensions

The mean ± SD was used to provide descriptive statistics for economic costs (Table 2). Annually, a user with dementia in a nursing home displays an average cost of €15.3 thousand (*SD* = 4021), while a user without dementia presents an expenditure of €12.3 thousand (*SD* = 1288), a difference of €3000 between the two types of subjects.

**Table 2:** **Economic costs by size and weight of the cost volume [mean (SD)]**.

**Dimension**	**Average annual cost/user with dementia (€)**	**%**	**Average annual cost/user without dementia (€)**	**%**
Emergency rooms or appointments, in public or private institutions	105 (123.8)	1	75 (80)	1
Days hospitalized	300 (571.6)	2	165 (336.3)	1
Medication	467 (518.4)	3	280 (273.7)	3
Social services used	11,161 (0.0)	73	11,161 (0.0)	91
Technical aids	651 (396.0)	4	138 (275.3)	1
Rehabilitation services	2604 (3773.4)	17	470 (1006.4)	4
Total cost	15,287 (4020.9)	100	12,289 (1287.8)	100

Social services are the largest share of the costs. The values of this dimension are the same for both groups, enough so that it is difficult to distinguish between them. In Portugal the financial support provided by the state is fixed, regardless of the economic situation and the degree of dependence of the user (Silva et al., 2015). However, at nursing homes, it can be given an additional value attributed by user in 2nd degree dependency situation (Silva et al., 2015). In the year of 2011–2012 this value was €65.35 per individual by month, and if the institution has more than 75% of users with this level of dependence was assigned an additional amount of €45.78 (Ministry of Solidarity Employment and Social Security and Union of Portuguese Mutual Societies, 2011).

For the dementia group, however, the use of social services represents 73% of the costs, while the same plot for subjects without dementia accounts for about 91% of the costs. After this portion, representing the largest volume in both groups are: rehabilitation services, technical aids, medication, hospitalization, and use of emergency services and hospital appointments at a private institution or a Health.

Except for the group without dementia, the size of the use of medication (with dementia: 467 €; without dementia: 280 €) takes up a larger volume than the use of technical aids (with dementia: 651 €; without dementia: 138 €). It should be noted that the size of technical aids includes the cost of diapers. The annual charge in diapers for the group with dementia was €19.5 thousand (*SD* = 3709), about 83% of the costs associated with technical aids. For the group of subjects without dementia, diapers accounted for about 69% of the cost of all technical aids.

### Predictive costs model

In the univariable general linear models for each of the 26 variables analyzed, considering a *p* < 0.1, eight significant variables were obtained. There are three types of significant variables. First, there are the communication variables (“plainness of speech” and “ability to make yourself understood”). There is a known relationship between dementia and lower control capabilities in the areas of verbal expression, auditory comprehension, repetition, reading, and writing (Murdoch et al., 1987). Communication problems lead to behavioral changes (Horner, 1985). To monitor and prevent problems associated with behavioral changes, it is necessary to increase vigilance on the part of the auxiliary personnel, which increases costs.

The second type of variable is related to the Activities of Daily Living. In this model, three variables were significant “self-performance of Activities of Daily Living” including: “locomotion outside the institution,” “dressing,” and “personal hygiene.”

The third type of variable is associated with the state of health concerning “thyroid disorders” and “risk for pressure ulcers.” There is a correlation between the prevalence of pressure ulcers and certain diseases, such as dementia (Tsokos et al., 2000). The presence of problems with nutritional status, chronic diseases, changes in sensory perception, functionality, and the immobility of people with dementia increases their vulnerability and likelihood of developing pressure sores (Oot-Giromini, 1993; Zekry et al., 2008; Jaul, 2010).

Preventing or reducing the likelihood of developing ulcers through methods such as frequent alternation decubitus, the use of specific support surfaces, or diapering in the case of incontinence and hydration, leads to an increase of time spent by the subjects with professionals, which translates into increased costs (European Pressure Ulcer Advisory Panel and American National Pressure Ulcer Advisory Panel, 2009). Thyroid clinical disorders are associated with cognitive disorders and dementia (Dugbartey, 1998). The costs associated with specific medication for this gland's problems were analyzed. All subjects take one pill per day, which represents an annual price of €43 (INFARMED, 2012) because there appears to be no specific medication for thyroid disorders, which increases costs. It was found, though, that the five subjects with thyroid problems combined to make a total of 56 days of hospitalization, which translates into €4760. Additionally, physiotherapy represented an annual cost of €24232, about €4846 per user. We could not clarify the origin of days of hospitalization and physical therapy sessions, but this may be the result of two factors. They may have arisen randomly, so there is not actually any link between the internment/physiotherapy and thyroid disorders, or in a spurious manner, in which there is a connection to the changes in the thyroid.

Other variables would also be expected to be significant. There may be several factors responsible for the insignificance of variables that would be expected as a result of the predictive variables costs. First, it is assumed that the groups can be very homogenous and there are no evident differences in the end. The fact stands that 50% of the subjects are members of the group without dementia, but the BIMS classify them as having moderate to severe cognitive impairment, which can also influence the results. As stated above this is quite common in nursing homes (Graham et al., 1997). The existence of only four individuals with moderate-stage dementia does not leave reliably comparable groups of people with dementia in the moderate and severe stages.

The eight variables of significance were entered into the multivariable general linear model (Table 3). Then the variables with the highest *p*-values were removed to obtain the final model (Table 4).

**Table 3:** **Multivariable general linear model of covariates and factors associated with economic costs (***n*** = 72)**.

**Variable**	**B (slope of the regression line)**	**95% confidence interval for B**	**Probability**
**CLARITY OF SPEECH**			
Clear speech	644.796	−1637.536; 2927.128	0.574
Slurred speech/no speech	−	−	
**ABILITY TO MAKE YOURSELF UNDERSTOOD**			
Understood	−3294.063	−5843.691; −744.435	0.012
Rarely/never understood	−	−	
**AUTO PERFORMANCE LOCOMOTION OUTSIDE THE INSTITUTION**			
Independent	−303.109	−1922.075; 1315.857	0.192
Total dependence	−3447.875	−7337.208; 441.458	
Activity did not occur	−	−	
**AUTO PERFORMANCE: PERSONAL HYGIENE**			
Independent	−252.235	−2562.575; 2058.106	0.828
Total dependence	−	−	
**AUTO PERFORMANCE: GETTING DRESSED**			
Independent	−2,693.293	−5112.769; −273.816	0.030
Total dependence	−	−	
**LIMITATIONS OF RANGE OF MOTION OF UPPER LIMBS**			
No deterioration	665.716	−2732.800; 4064.232	0.116
Deterioration of one side	3551.739	−756.317; 7859.796	
Impairment of both sides	−	−	
**DISTURBANCES IN THYROID**			
No	−3292.521	−6448.073; −136.969	0.041
Yes	−	−	
**RISK OF PRESSURE ULCERS**			
No	−793.720	−4559.510; 2972.070	0.675
Yes	−	−	

**Table 4:** **Final Model of covariates and factors associated with economic costs (***n*** = 72)**.

**Variable**	**B (slope of the regression line)**	**95% confidence interval for B**	**Probability**
**ABILITY TO MAKE YOURSELF UNDERSTOOD**			
Understood	−2442.192	−4277.343; −607.040	0.010
Rarely/never understood	–	–	
**AUTO PERFORMANCE: GETTING DRESSED**			
Independent	−2477.784	−4161.358; −794.210	0.005
Total dependence	–	–	
**DISTURBANCES IN THYROID**			
No	−3197.691	−5,890.957; −504.425	0.021
Yes	–	–	

Individuals who are understood verbally influence lower costs as compared to subjects who rarely/are never understood. The subjects who rely totally on others to dress them positively influences costs and those who have thyroid problems exert a positive influence on increased costs. Considering the variables that make up the final model interactions between the three variables (two by two), predictive costs were also tested. Since none of the interactions proved statistically significant, it was decided to not include any of this information in the final model.

In models created the socio-demographic variables, as age and gender, were not found to be significant in the variable costs.

## Discussion

This study is the first in Portugal to analyze the direct costs of dementia in nursing homes. A user in a nursing home with dementia presents a cost of €15,287, about €3 thousand more than the cost of a user without dementia in the same service. The prevalence of people with dementia in the four non-profit organizations analyzed is 17%. The latest data shows that in 2010 about 66,275 people lived in nursing homes (Letra and Martín, 2010). At a rate of 17%, it estimated that in Portugal alone there exist 11,267 people in nursing homes with a diagnosis or signs of dementia. Given the figures, 11,267 diagnosed people represent an annual cost of €172 million. This represents 0.104% of the Portuguese GDP in the year 2012 (FFMS, 2013).

Given that there is no common methodology between the present and previous studies (Wimo et al., 2007, 2010), a comparison of data is not reliable, but it has been presented to verify discrepancies.

In 2005, the direct cost with 113,039 people was around €736 million (Wimo et al., 2007). Considering the values of the present study, with regard to 113,039 people, the costs would accrue to €992 million according to the value calculated (Wimo et al., 2007).

There are several alternatives to the institutionalization of a person with dementia that would result in lower annual costs than those that were found. We present three policies to support families in preventing hospitalization through specialization or a combination of services: the use of (1) day centers specific to dementia, (2) home care services along with a location tracking device, and (3) respite care services.

Specific day centers offer appropriate care, therapies, and activities catered to their patients that stimulate their participation and integration. Private sector in Madrid has specific day centers for Alzheimer's disease and other forms of dementia. On average, the monthly payment can vary between €175 and €935 (depending on schedules and contracted services) (Maria Wolff Alzheimer, 2013^2^). Comparatively, a nursing home in Madrid has a monthly cost of €1790 (Inforesidencias, 2012).

The second hypothesis is the combination between a home support service and a location tracking device. The monthly amount considered for the home support service (maximum reimbursement for the home support service is expressed in the Protocol of Cooperation 2011–2012) is €239 (Ministry of Solidarity Employment and Social Security and Union of Portuguese Mutual Societies, 2011). In Portugal, the acquisition system for a portable locating device with GPS/LBS is around €150 for the equipment, plus a €20 monthly fee (Portuguese Red Cross, 2013). This combination of services represents an annual cost of €3284.

The last alternative is respite care services. The total cost of implementing a group psychoeducational intervention of 11 sessions with an average of nine participants per session is €4123 (Caravau et al., 2013). This amounts to around €458 per participant, with a payback period of 8 years at €5 per month.

The predictive model shows that only three of the variables led to increased costs of internment with subjects in nursing homes. Subjects who rarely or are never understood, those who need support in getting dressed, and those with clinical disorders of the thyroid are showing greater cost shares for inpatient care. It was appropriate that in future investigations were analyzed the relationship between the economic costs to the degree of dependence of the person. However, in this study was not possible to do so because it would be necessary to establish a minimum number of people for each group, about 20–30 people per group. There was no statistical capacity with this sample for this procedure.

The costs of caring for a person with dementia are high and represent a major burden on the economy of the country, patients, and families. Knowing the real costs of an individual with dementia in nursing homes allows for a more effective management and efficient use of national resources.

The present results may support the evaluation of the impact of policies, the prioritization of expenditures, and the adjustment of decisions based on the Portuguese reality.

Overall, it is believed that this study has obtained a good approximation of the actual costs of a user with dementia. Studies such as this should be applied at a national level to fully understand a panoramic view of Portugal, and in future research it would be interesting to analyze the cost that represented the use of social resources to the Government, to the patient and the family.

### Conflict of interest statement

The authors declare that the research was conducted in the absence of any commercial or financial relationships that could be construed as a potential conflict of interest.

## Footnotes

^1^Portaria n.° 132/2009 de 30 de Janeiro. *Diário da República, 1*.^a^ *série—N*. ° *21*. Lisboa: Ministério da Saúde.

^2^Maria Wolff Alzheimer. (2013). Available online at: http://mariawolff.info/.
